# Endothelium-dependent relaxation of rat aorta to a histamine H_3_ agonist is reduced by inhibitors of nitric oxide synthase, guanylate cyclase and Na^+^,K^+^-ATPase

**DOI:** 10.1155/S0962935196000129

**Published:** 1996-02

**Authors:** D. M. Djuric, M. T. Nesic, I. Z. Andjelkovic

**Affiliations:** 1 Institute of Physiology Medical Faculty University of Belgrade Visegradska 26/II P.O. Box 783 Belgrade 11000 Serbia

## Abstract

The possible involvement of different effector systems (nitric oxide synthase, guanylate cyclase, β-adrenergic and muscarinic cholinergic receptors, cyclooxygenase and lipoxygenase, and Na^+^,K^+^-ATPase) was evaluated in a histamine H_3_ receptor agonist-induced ((R)α-methylhistamine, (R)α-MeHA) endothelium-dependent rat aorta relaxation assay. (R)α-MeHA (0.1 nM – 0.01 mM) relaxed endothelium-dependent rat aorta, with a pD_2_ value of 8.22 ± 0.06, compared with a pD_2_ value of 7.98 ± 0.02 caused by histamine (50% and 70% relaxation, respectively). The effect of (R)α-MeHA (0.1 nM – 0.01 mM) was competitively antagonized by thioperamide (1, 10 and 30 nM) (pA_2_ = 9.21 ± 0.40; slope = 1.03 ± 0.35) but it was unaffected by pyrilamine (100 nM), cimetidine (1 μM), atropine (10 μM), propranolol (1 μM), indomethacin (10 μM) or nordthydroguaiaretic acid (0.1 mM). Inhibitors of nitric oxide synthase, L-N^G^-monomethylarginine (L-NMMA, 10 μM) and N^G^-nitro-L-arginine methylester (L-NOARG, 10 μM) inhibited the relaxation effect of (R)α-MeHA, by approximately 52% and 70%, respectively). This inhibitory effect of L-NMMA was partially reversed by L-arginine (10 μM). Methylene blue (10 μM) and ouabain (10 μM) inhibited relaxation (R)α-MeHA-induced by approximately 50% and 90%, respectively. The products of cyclooxygenase and lipoxygenase are not involved in (R)α-MeHA-induced endothelium-dependent rat aorta relaxation nor are the muscarinic cholinergic and β-adrenergic receptors. The results also suggest the involvement of NO synthase, guanylate cyclase and Na^+^,K^+^-ATPase in (R)α-MeHA-induced endothelium-dependent rat aorta relaxation.


Mediators of Inflammation 5, 69–74 (1996)

### Endothelium-dependent relaxation of rat aorta to a histamine $H_3$ agonist is reduced by inhibitors of nitric oxide synthase, guanylate cyclase and $Na^+$,$K^+$-ATPase

D. M. Djuric,[CA] M. T. Nesic and I. Z. Andjelkovic

Institute of Physiology, Medical Faculty University of Belgrade, Visegradska 26/II, P.O. Box 783, 11000 Belgrade, Yugoslavia.
Fax: (+381) 11 644263.

[CA]Corresponding Author

**The possible involvement of different effector systems (nitric oxide synthase, guanylate cyclase, β-adrenergic and muscarinic cholinergic receptors, cyclooxygenase and lipoxygenase, and $Na^+$,$K^+$-ATPase) was evaluated in a histamine $H_3$ receptor agonist-induced ((*R*)α-methylhistamine, (*R*)α-MeHA) endothelium-dependent rat aorta relaxation assay. (*R*)α-MeHA (0.1 nM - 0.01 mM) relaxed endothelium-dependent rat aorta, with a $pD_2$ value of $8.22 \pm 0.06$, compared with a $pD_2$ value of $7.98 \pm 0.02$ caused by histamine (50% and 70% relaxation, respectively). The effect of (*R*)α-MeHA (0.1 nM - 0.01 mM) was competitively antagonized by thioperamide (1, 10 and 30 nM) ($pA_2 = 9.21 \pm 0.40$; slope = $1.03 \pm 0.35$) but it was unaffected by pyrilamine (100 nM), cimetidine (1 μM), atropine (10 μM), propranolol (1 μM), indomethacin (10 μM) or nordihydroguaiaretic acid (0.1 mM). Inhibitors of nitric oxide synthase, L-$N^g$-monomethylarginine (L-NMMA, 10 μM) and $N^g$-nitro-L-arginine methylester (L-NOARG, 10 μM) inhibited the relaxation effect of (*R*)α-MeHA, by approximately 52% and 70%, respectively). This inhibitory effect of L-NMMA was partially reversed by L-arginine (10 μM). Methylene blue (10 μM) and ouabain (10 μM) inhibited relaxation (*R*)α-MeHA-induced by approximately 50% and 90%, respectively. The products of cyclooxygenase and lipoxygenase are not involved in (*R*)α-MeHA-induced endothelium-dependent rat aorta relaxation nor are the muscarinic cholinergic and β-adrenergic receptors. The results also suggest the involvement of NO synthase, guanylate cyclase and $Na^+$,$K^+$-ATPase in (*R*)α-MeHA-induced endothelium-dependent rat aorta relaxation.**

**Key words:** Aorta, Endothelium, Guanylate cyclase, Histamine $H_3$ receptor, $Na^+$,$K^+$-ATPase, Nitric oxide synthase

### Introduction

Histamine is present in essentially all tissues and it can stimulate all three classes of histamine receptors. It is found in significant concentrations in the blood and also in the vessel walls.[1] It has been known for several years that histamine receptor subtypes vary in different isolated vascular tissues, depending upon the anatomic location, species and physiological response. It is known that two types of histamine receptors, $H_1$ and $H_2$, participate in vascular responses to histamine.[2] Intravascular administration of histamine elicits a concentration-dependent fall in blood pressure in most species. Many studies have indicated the involvement of histamine $H_1$ and $H_2$ receptors in this depressor response.[3] The histamine $H_1$ and $H_2$ receptor-mediated actions of histamine on effector cells are linked with the accumulation of cGMP, inositol phospholipids and cAMP, respectively.[4–6] In different blood vessels cGMP formation is activated by endothelium-derived relaxing factor (EDRF)[7] while cAMP formation is stimulated by prostacyclin ($PGI_2$).[8,9]

The histamine $H_3$ receptors were found within the central nervous system of the rat and the human where they appear to be involved in the feedback control of both histamine synthesis and release of the level of histaminergic nerve endings.[10,11] Furthermore, stimulation of histamine $H_3$ receptors has been shown to inhibit adrenergic and cholinergic neurotransmission in the peripheral autonomic nervous system.[12,13] There is some controversy about whether histamine $H_3$ receptors are present on the sympathetic nerve fibres innervating blood vessels.[12] Stimulation of histamine $H_3$ receptors by specific agonist mediated vascular relaxant effects is

© 1996 Rapid Science Publishers caused by mechanism(s) which are not clear at present. In two isolated vessels, the rabbit middle cerebral artery[14,15] and guinea-pig aorta[16] a potent and selective histamine $H_3$ agonist, (*R*)α-methylhistamine ((*R*)α-MeHA), caused relaxation probably via stimulation of postsynaptic histamine $H_3$ receptors. These findings suggest that, depending on the species and the experimental model, several mechanisms (activation of pre- and post-synaptic histamine $H_3$ receptors or histamine $H_3$ receptor-independent mechanisms) contribute to the overall effect of (*R*)α-MeHA on cardiovascular function.[17]

In our previous report the existence of histamine $H_3$ receptors on rat aorta endothelium was shown.[18] The present study was therefore undertaken to assess the possible role of histamine $H_3$ receptors located on the rat aorta endothelium and their interactions with other effector systems.

### Materials and Methods

*Vascular preparations:* Male Wistar rats weighing between 100 and 200 g were stunned and the thoracic aorta was excised and dissected free of surrounding tissue. Ring segments (4 mm) were prepared and fixed isometrically in a 20 ml organ bath containing Tyrode's solution of the following composition (mM): NaCl, 136.9; KCl, 2.69; $CaCl_2$, 1.8; $MgCl_2$, 1.05; $NaHCO_3$, 11.9; $NaH_2PO_4$, 0.42; and glucose 5.55, at 37°C under a moderate tension of 1 g for 90 min (the optimal point of its length–tension curve as determined from the tension developed in response to potassium chloride 40 mM) and gassed with 95% $O_2$/5% $CO_2$. The preparations were precontracted by phenylephrine (300 nM). In some preparations the endothelium was removed mechanically by gentle and careful rubbing of the intimal surface with a stainless-steel wire (31-gauge diameter) in order to avoid stretching and damaging the vascular smooth muscle cells. The presence of endothelium was confirmed by using acetylcholine (300 nM). The failure of acetylcholine to induce relaxation of preparations was taken as an indication of endothelium removal.

*Experimental procedure:* After the equilibration period, concentration–response curves were obtained by cumulative addition of histamine (0.1 nM – 0.01 mM) or (*R*)α-MeHA (0.1 nM – 0.01 mM) on precontracted preparations alone or in the presence of pyrilamine (100 nM), cimetidine (1 μM) and thioperamide (1 nM, 10 nM, 30 nM) for (*R*)α-MeHA.

All drugs were added directly to the bath in a volume of 150 μl and the concentrations given are the calculated final concentrations in the bath solution. When potassium chloride was used as a spasmogen, the stated concentration exluded the potassium chloride already present in Tyrode's solution.

*Data analysis:* Responses are expressed as a percentage of the maximal relaxation induced by papaverine (100%, 0.1 mM). The slope of the log concentration–response curve, correlation coefficient ($r$), $E_{max}$ (maximum response) and $pA_2$ (−log molar concentrations of antagonist reducing the agonist response by a factor of 2) values were evaluated from concentration–response curves plotted for (*R*)α-MeHA in the presence of thioperamide. For calculating these different values the data are expressed as means ± S.E.M.; $n$ refers to the number of experiments. $E_{max}$ values were compared using Student's $t$-test. $p$ values less than 0.05 were considered to be significant.

*Drugs:* The following compounds were used: acetylcholine chloride (Sigma), phenylephrine hydrochloride (Sigma), histamine dihydrochloride (Sigma), (*R*)α-methylhistamine (Research Biochemicals Incorporated), pyrilamine maleate (Sigma), cimetidine (Sigma), thioperamide maleate (Research Biochemicals Incorporated), atropine sulphate (Sigma), propranolol hydrochloride (Sigma), L-$N^g$-monomethylarginine (L-NMMA, Research Biochemicals Incorporated), $N^g$-nitro-L-arginine methylester (L-NOARG, Research Biochemicals Incorporated), L-arginine (Sigma), indomethacin (Sigma), nordihydroguaiaretic acid (NDHGA, Sigma), ouabain octahydrate (Serva), methylene blue (Sigma) and papaverin hydrochloride (Sigma). All solutions were kept on ice until use except thioperamide which was dissolved in dimethylsulphoxide (previous experiments had shown that the solvents used had no effects on the preparations) and (*R*)α-MeHA which was diluted in water and stored as an aliquot (100 μl) at −20°C. Indomethacin was dissolved in an equimolar concentration with $Na_2CO_3$.

### Results

***The influence of endothelium on responses to histamine and* (R)α*-MeHA:*** Histamine (0.1 nM – 0.01 mM) induced concentration-dependent relaxation of phenylephrine (300 nM)-precontracted rat aorta with intact endothelium, reaching approximately 70% of the papaverine-induced maximum relaxation (0.1 mM) ($pD_2 = 7.98 \pm 0.02$). Removal of the endothelium abolished the relaxation to histamine.

The potent and selective histamine $H_3$ agonist,

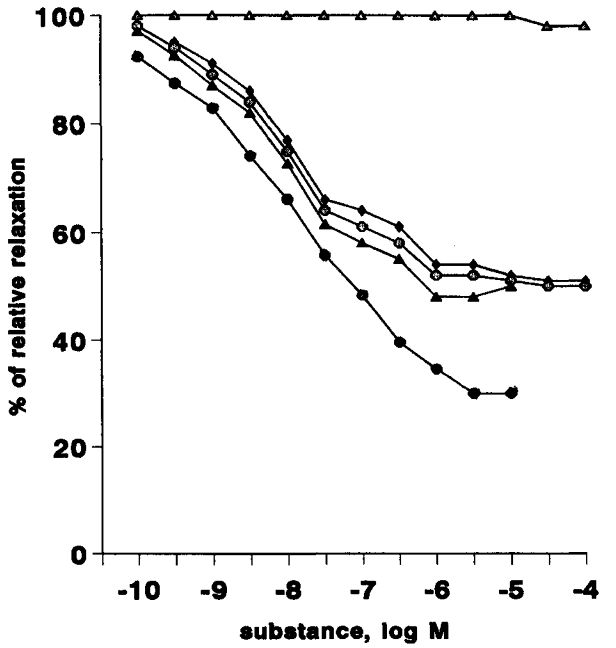

FIG. 1. Concentration-response curves for histamine and (*R*)α-MeHA in rat aorta with intact endothelium ((*R*)α-MeHA alone or in the presence of pyrilamine or cimetidine). The data are expressed as means ($n = 6$) (S.E.M. are exluded from diagram for clarity and do not exceed 15% of the mean value for each point). ▲, (*R*)α-MeHA; ●, histamine; ◎, pyrilamine (100 nM); ◆, cimetidine (1 μM); △, endothelium denuded.

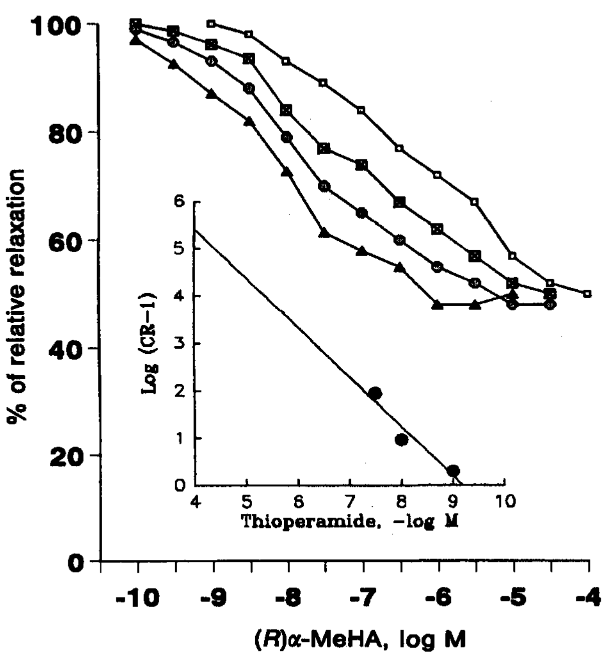

FIG. 2. Concentration-response curves for (*R*)α-MeHA in rat aorta with intact endothelium alone or in the presence of thioperamide. The data are expressed as means ($n = 6$) (S.E.M. are exluded from diagram for clarity and do not exceed 15% of the mean value for each point). The intercept on the abscissa scale gives the $pA_2$ value; $y = -1.04x + 9.55$, $r = 0.948$, $pA_2 = 9.21 \pm 0.40$, slope $1.03 \pm 0.35$, for thioperamide. ▲, Control; ◎, thioperamide, 1 nM; ⊠, thioperamide, 10 nM; □, thioperamide, 30 nM.

(*R*)α-MeHA (0.1 nM – 0.01 mM) induced concentration-dependent relaxation of phenylephrine (300 nM)-precontracted rat aorta with intact endothelium reaching approximately 50% of the papaverine-induced maximum relaxation (0.1 mM) ($pD_2 = 8.22 \pm 0.06$). Removal of the endothelium abolished the relaxation to (*R*)α-MeHA (Fig. 1).

*Effects of pyrilamine, cimetidine and thioperamide:* Pyrilamine (100 nM, $K_d$ for $H_1$ = 0.8 nM, $K_d$ for $H_2$ = 5.2 μM, $K_d$ for $H_3$ = > 3 μM, see Hill[19]) or cimetidine (1 μM, $K_d$ for $H_2$ = 0.8 μM, $K_d$ for $H_1$ = 0.45 mM, $K_d$ for $H_3$ = 33 μM, see Hill[19]) did not influence (*R*)α-MeHA-induced endothelium-dependent rat aorta relaxation (Fig. 1).

When thioperamide (1 nM, 10 nM, 30 nM, $K_d$ for $H_1$ > 100 μM, $K_d$ for $H_2$ > 10 μM, $K_d$ for $H_3$ = 4.3 nM, see Arrang *et al.*[11]) was present the concentration–response curve for (*R*)α-MeHA-induced relaxation was shifted to the right without a significant change of the $E_{max}$. Schild plot analysis indicated that antagonism by this compound was competitive. The slope for the regression curve was $1.03 \pm 0.35$ with a $pA_2$ value of $9.21 \pm 0.40$ (Fig. 2).

*Effects of atropine, propranolol, indomethacin and NDHGA:* Neither atropine (10 μM), propranolol (1 μM), indomethacin (10 μM) nor NDHGA (0.1 mM) significantly reduced (*R*)α-MeHA-induced endothelium-dependent rat aorta relaxation (data not shown).

*Inhibition by L-NMMA and L-NOARG:* Both inhibitors of NO synthase inhibited (*R*)α-MeHA-induced endothelium-dependent relaxation in a concentration-dependent manner and this effect was maximal after incubation with 10 μM L-NMMA or 10 μM L-NOARG (approximately 52% and 70% inhibition, respectively). The addition of L-arginine (10 μM) partly reversed L-NMMA-induced inhibition of the response to (*R*)α-MeHA (Fig. 3). Higher concentrations (> 10 μM) of both inhibitors induced no further reduction of relaxation (not shown).

*Inhibition by methylene blue and ouabain:* Methylene blue (10 μM) inhibited the (*R*)α-MeHA-induced endothelium-dependent rat aorta relaxation (approximately 50% inhibition). Higher concentrations (> 10 μM) of inhibitor induced no further reduction of relaxation. Ouabain (10 μM) inhibited the (*R*)α-MeHA-induced endothelium-dependent relaxation (approximately 90% inhibition) (Fig. 4). Higher concentrations (> 10 μM) of inhibitor induced no further reduction of relaxation.

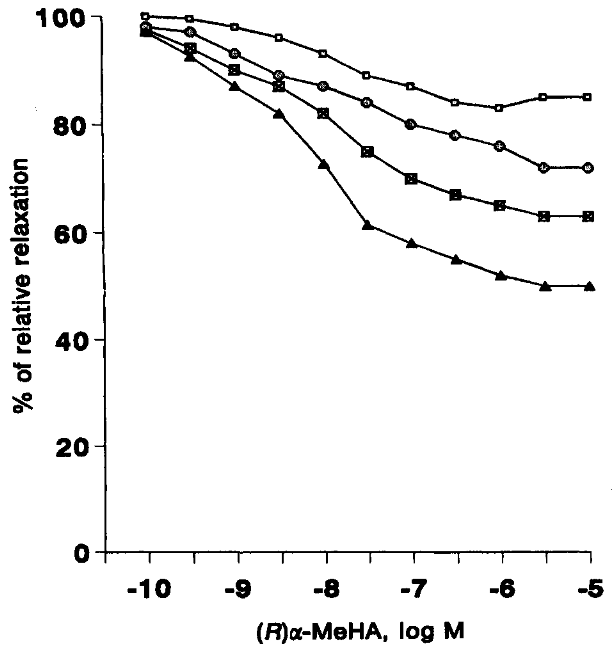

FIG. 3. Concentration–response curves for (R)α-MeHA in rat aorta with intact endothelium in the presence of L-NMMA, L-NMMA + L-arginine or L-NOARG. The data are expressed as means ($n=6$) (S.E.M. are exluded from diagram for clarity and do not exceed 15% of the mean value for each point). ▲, Control; ◎, L-NMMA, 10 μM; ⊠, L-NMMA + L-Arg (10 μM); □, L-NOARG, 10 μM.

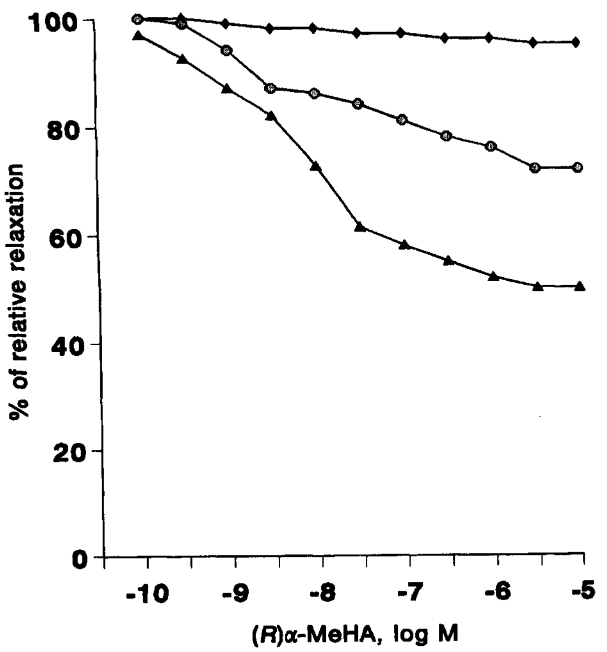

FIG. 4. Concentration–response curves for (R)α-MeHA in rat aorta with intact endothelium in the presence of methylene blue or ouabain. The data are expressed as means ($n=6$) (S.E.M. are excluded from diagram for clarity and do not exceed 15% of the mean value for each point). ▲, Control; ◎, methylene blue, 10 μM; ◆, ouabain, 10 μM.

### Discussion

The novel histamine $H_3$ receptors were identified as inhibitory presynaptic autoreceptors on histamine-containing nerve terminals in the rat brain cortex but they have since been shown to inhibit the release of various neurotransmitters both in the central and peripheral nervous system.[19] Recent articles provide strong evidence for the presence of histamine $H_3$ receptors at different sites, including rabbit middle cerebral artery endothelium,[14,15] guinea-pig aorta,[16] mesenteric artery,[12] rabbit saphenous artery,[20] guinea-pig myocardium,[21] guinea-pig ileum,[22] guinea-pig lung and bronchiole,[23,24] guinea-pig intestine,[25] porcine small intestine,[26] rabbit gastric glands,[27] human adenoidal mast cells,[28] and human and rhesus monkey brain.[29] The endothelium-dependent relaxation to histamine (0.1 nM – 0.01 mM) was competitively antagonized by pyrilamine (1 nM, 7 nM, 10 nM) with a $pA_2$ value of $9.336 \pm 0.34$ and a slope of $1.09 \pm 0.36$.[18] It was also competitively antagonized by thioperamide (1 nM, 10 nM, 30 nM) with a $pA_2$ value of $9.31 \pm 0.16$ and a slope of $0.94 \pm 0.10$, but it was unaffected by cimetidine (1 μM).[18] The results with both histamine and pyrilamine suggest the presence of histamine $H_1$ receptors on rat aorta endothelium, which is in agreement with results of other authors.[30–32]

Thioperamide antagonizes both histamine and (R)α-MeHA-induced relaxations resulting in about the same $pA_2$ values (9.31 for histamine and 9.21 for (R)α-MeHA in present paper, respectively). These $pA_2$ values are close to that (8.96) found for blockade of histamine $H_3$ mediated inhibition of [$^3$H]-histamine release in rat brain slices.[11] The $pA_2$ value of the histamine $H_3$ antagonist thioperamide was very similar to its values for various responses mediated by histamine $H_3$ receptors.[33] A major point is the finding that histamine is more active than (R)α-MeHA, which makes it uncertain whether only histamine $H_3$ receptors are involved. There is a heterogenous population of histamine receptors, $H_1$ and $H_3$, on rat aorta endothelium.[18] This is not surprising although the known histamine vascular effects in different biological species involve two receptor systems, the histamine $H_1$ and histamine $H_2$ receptors. New observations suggest that histamine $H_3$ receptors are also localized at the postsynaptic level in rabbit middle cerebral artery endothelium,[14,15] guinea-pig aorta[16] and in the epithelial wall of guinea-pig bronchioles,[23] and act on the smooth muscle. The mechanism underlying the inhibitory effect of histamine $H_3$ receptor stimulation on neurotransmitter release in central and peripheral tissues remains to be established. However, comparison with other receptor systems, known to have a similar effect on neurotransmitter release (e.g. adenosine $A_1$ receptor, opiate receptor and $\alpha_2$ adrenoceptor) suggests a number of possibilities for the effector systems linked to the histamine $H_3$ receptor. The possibilities include inhibition of adenylate cyclase activity, activation of $K^+$ channels and inhibition of voltage-dependent $Ca^{2+}$ channels.[34,35] Studies in slices of guinea-pig hippocampus have shown that the histamine $H_3$ agonist (*R*)α-MeHA is not able to inhibit dimaprit-induced cAMP accumulation[36] suggesting that histamine $H_3$ receptors are not negatively linked to adenylate cyclase. It is known that (*R*)α-MeHA, a potent and selective histamine $H_3$ agonist, induced endothelium-dependent relaxation of high-$K^+$ precontracted rabbit middle cerebral artery. That relaxation was not affected by β-adrenoceptors, muscarinic or dopamine receptor antagonists.[15] Our results with atropine and propranolol also showed that neither muscarinic cholinergic receptors nor β-adrenoceptors were involved in (*R*)α-MeHA-induced endothelium-dependent rat aorta relaxation.

The identification of nitric oxide (NO) (or a compound containing the NO ligand) such as EDRF[37,38] and the finding that L-NMMA or L-NOARG are inhibitors of NO synthesis in vascular endothelium[39,40] have emphasized the importance of local control of vasomotor tone. Nitric oxide enhances production of cGMP in vascular smooth muscles through activation of soluble guanylate cyclase, which in turn activates $Ca^{2+}$-ATPase to reduce intracellular $Ca^{2+}$ concentration and induces muscle relaxation. It has been demonstrated that L-NAME (a competitive inhibitor of NO synthesis[41]), specifically inhibits cGMP formation due to the activation of muscarinic, histamine, bradykinin and neurotensin receptors in mouse neuroblastoma N1E-115 cells.[42] In our experiments the inhibitory effects of L-NMMA and L-NOARG on (*R*)α-MeHA-induced endothelium-dependent rat aorta relaxation were also observed, indicating that these substances inhibit NO formation by competing with L-arginine for binding to NO synthase. The addition of L-arginine partly reversed L-NMMA-induced inhibition of response. We also found that the inhibitory effect of L-NOARG was greater than that of L-NMMA (70% and 52%, respectively) which is in agreement with the observations of different authors.[15,43,44] Methylene blue (which blocks guanylate cyclase[45]) prevented the increase in cGMP and thus inhibited (*R*)α-MeHA-induced endothelium-dependent rat aorta relaxation.

The decrease with age in the dilator response of rat thoracic aorta to histamine is due to a decreased cGMP production and to an age-dependent decrease in endothelium function.[46] Also, in guinea-pig airway smooth muscle histamine can activate nitric oxide synthase resulting in the release of NO.[47]

In different blood vessels, cGMP formation is activated by EDRF,[7] while cAMP formation is stimulated by $PGI_2$.[8,9] Particularly from experiments with endothelial cells cultured *in vitro* several classes of agonist have been demonstrated to interact with surface receptors leading to $PGI_2$ synthesis. These include proteins and peptides (thrombin and bradykinin), amines (histamine at $H_1$ receptors), eicosanoids (leukotriene $C_4$) and purines (ATP and ADP).[48] (*R*)α-MeHA-induced endothelium-dependent rabbit middle cerebral artery relaxation was partially reduced by tranylcypromine (an inhibitor of leukotriene synthesis) and also inhibited by dexamethasone (an inhibitor of prostaglandin synthesis probably at the level of phospholopase $A_2$[49]) and indomethacin (an inhibitor of $PGI_2$ synthesis[50]) indicated that a prostanoid (probably $PGI_2$) could also be involved in (*R*)α-MeHA-induced endothelium-dependent rabbit middle cerebral artery relaxation.[15] The implication of results with indomethacin and NDHGA is that (*R*)α-MeHA does not induce the release of the relaxing factors containing metabolites of arachidonic acid via cyclooxygenase (e.g. $PGE_1$, $PGE_2$, $PGI_2$) or via lipoxygenase (leukotrienes) on rat aorta endothelium.

In many arteries, the release of EDRF by acetylcholine (Ach) is accompanied by an endothelium-dependent hyperpolarization, while EDHF is the possible mediator.[51] The EDHF-induced hyperpolarization is produced by an increased permeability of the membrane to $K^+$ ions with no change in either cAMP or cGMP.[52] It has also been concluded that the hyperpolarization resulted from stimulation of $Na^+,K^+$-ATPase. It has been observed that in rat aorta and rat main pulmonary artery ACh released two different substances from endothelium. On factor (EDRF) is responsible for vascular muscle relaxation while the other (EDHF) hyperpolarizes the muscle membrane by opening $^{86}Rb$-permeable $K^+$ channels.[51] However, ouabain (an inhibitor of the $Na^+,K^+$-ATPase) inhibited (*R*)α-MeHA-induced endothelium-dependent rat aorta relaxation much more than L-NOARG (90% and 70% inhibition, respectively). It means that in addition to NO, some factor other than NO (e.g. EDHF) could participate in (*R*)α-MeHA-induced endothelium-dependent rat aorta relaxation. Inhibition of the $Na^+,K^+$-ATPase by ouabain would shift the membrane potential to a less negative level and thus reduce or abolish any hyperpolarization associated with $K^+$ channel opening.

In conclusion, we suggest that the products of cyclooxygenase and lipoxygenase are not involved in ($R$)α-MeHA-induced endothelium-dependent rat aorta relaxation in addition to muscarinic cholinergic and β-adrenergic receptors. The results also suggest the involvement of NO synthase, guanylate cyclase and $Na^+$,$K^+$-ATPase in histamine $H_3$ receptor agonist-induced (($R$)α-MeHA) endothelium-dependent rat aorta relaxation.

### References

1. Ryan MS, Brody MS. Neurogenic and vascular stores of histamine in the dog. *J Pharmacol Exp Ther* 1972; **181:** 183–186.
2. Konishi M, Toda N, Yamamoto M. Different mechanisms of action of histamine in isolated arteries of the dog. *Br J Pharmacol* 1981; **74:** 111–118.
3. Owen DAA. Histamine receptors in the cardiovascular system. *Gen Pharmacol* 1977; **8:** 141–144.
4. Schwartz JC, Garbarg M, Pollard H. Histaminergic transmission in the brain. In: Bloom FF, Mountcastele VB, Geiger SR, eds. *Handbook of Physiology.* Bethesda, American Physiological Society (Section 1), 1986; **4:** 257–316.
5. Daum PR, Downes CP, Young JM. Histamine-induced inositol phospholipid breakdown mirrors $H_1$-receptor density in brain. *Eur J Pharmacol* 1983; **87:** 497–498.
6. Green JP. Histamine receptors. In: Meltzer HJ, ed. *Psychopharmacology: the third generation of progress.* New York, Raven Press, 1987, 273–279.
7. Rapoport RM, Murad F. Agonist induced endothelium-dependent relaxation in rat thoracic aorta may be mediated through cyclic GMP. *Circ Res* 1983; **52:** 352–357.
8. Gorman RR, Bunting S, Miller OV. Modulation of human platelet adenylate cyclase by prostacyclin (PGX). *Prostaglandins* 1977; **13:** 377–388.
9. Tateson JE, Moncada S, Vane JR. Effects of prostacyclin (PGX) on cyclic AMP concentrations in human platelets. *Prostaglandins* 1977; **13:** 389–397.
10. Arrang JM, Garbarg M, Schwartz JC. Auto-inhibition of brain histamine release mediated by a novel class ($H_3$) of histamine receptor. *Nature* 1983; **302:** 832–837.
11. Arrang JM, Garbarg M, Lancelot JC, *et al.* Highly potent and selective ligands for histamine $H_3$-receptors. *Nature* 1987; **327:** 117–123.
12. Ishikawa S, Sperelakis N. A novel class ($H_3$) of histamine receptors on perivascular nerve terminals. *Nature* 1987; **327:** 158–160.
13. Ichinose M, Stretton CD, Schwartz JC, Barnes PJ. Histamine $H_3$-receptors inhibit cholinergic neurotransmission in guinea-pig airways. *Br J Pharmacol* 1989; **97:** 13–16.
14. Ea-Kim L, Oudart N. A highly potent and selective $H_3$-agonist relaxes rabbit middle cerebral artery, *in vitro. Eur J Pharmacol* 1988; **150:** 393–396.
15. Ea-Kim L, Javellaud J, Oudart N. Endothelium-dependent relaxation of rabbit middle cerebral artery to a histamine $H_3$-agonist is reduced by inhibitors of nitric oxide and prostacyclin synthesis. *Br J Pharmacol* 1992; **105:** 103–106.
16. Rosic M, Collis SC, Andjelkovic I, Segal M, Djuric D, Zlokovic B. The effects of (R)alpha-methylhistamine on the isolated guinea pig aorta. In: Timmerman H, Van der Goot H, eds. *New Perspectives in Histamine Research,* Basel, Boston, Berlin: Birkhauser Verlag, 1991; 283–287.
17. Malinowska B, Schlicker E. $H_3$ receptor-mediated inhibition of the neurogenic vasopressor response in pithed rats. *Eur J Pharmacol* 1991; **205:** 307–310.
18. Djuric D, Andjelkovic I. The evidence for histamine $H_3$ receptor-mediated endothelium-dependent relaxation in isolated rat aorta. *Mediators of Inflammation* 1995; **4(3):** 217–221.
19. Hill JS. Distribution, properties and functional characteristics of three classes of histamine receptor. *Pharmacol Rev* 1990; **42:** 45–83.
20. Oike M, Kitamura K, Kuriyama H. Histamine $H_3$-receptor activation augments voltage-dependent $Ca^{2+}$ current via GTP hydrolysis in rabbit saphenous artery. *J Physiol (London)* 1992; **448:** 133–152.
21. Luo XX, Tan YH, Sheng BH. Histamine $H_3$-receptors inhibit sympathetic neurotransmission in guinea pig myocardium. *Eur J Pharmacol* 1991; **204(3):** 311–314.
22. Hew RWS, Hodgkinson SR, Hill SJ. Characterization of histamine $H_3$-receptors in guinea pig ileum with $H_3$-selective ligands. *Br J Pharmacol* 1990; **101:** 621–624.
23. Burgaud JL, Javellaud J, Oudart N. Bronchodilator action of an agonist for histamine $H_3$-receptors in guinea pig perfused bronchioles and lung parenchymal strips. *Lung* 1992; **170(2):** 95–108.
24. Burgaud JL, Oudart N. Bronchodilatation of guinea-pig perfused bronchioles induced by the $H_3$-receptor for histamine: role of epithelium. *Br J Pharmacol* 1993; **109:** 960–966.
25. Coruzzi G, Poli E, Bertaccini G. Histamine receptors in isolated guinea pig duodenal muscle: $H_3$-receptors inhibit cholinergic neurotransmission. *J Pharmacol Exp Ther* 1991; **258(1):** 325–331.
26. Schworer H, Katsoulis S, Racke K. Histamine inhibits 5-hydroxytryptamine release from porcine small intestine: involvement of $H_3$ receptors. *Gastroenterology* 1992; **102(6):** 1906–1912.
27. Bado A, Moizo L, Laigneau JP, Lewin MJ. Pharmacological characterization of histamine $H_3$ receptors in isolated rabbit gastric glands. *Am J Physiol* 1992; **262(1 Pt 1):** G56–61.
28. Bent S, Fehling U, Braam U, Schunack W, Schmutzler W. The influence of $H_1$-, $H_2$- and $H_3$-receptors on the spontaneous and ConA induced histamine release from human adenoidal mast cells. *Agents Actions* 1991; **33(1–2):** 67–70.
29. Martinez-Mir MI, Pollard H, Moreau J, *et al.* Three histamine receptors (H1, H2 and H3) visualized in the brain of human and non-human primates. *Brain Res* 1990; **526(2):** 322–327.
30. Van de Voorde J, Leusen I. Role of the endothelium in the vasodilator response of rat thoracic aorta to histamine. *Eur J Pharmacol* 1983; **87:** 113–120.
31. Van de Voorde J, Leusen I. Effect of histamine on aorta preparations of different species. *Arch Int Pharmacodyn* 1984; **268:** 95–105.
32. Carrier OG, White ER, Kirby LM. Histamine-induced relaxation of rat aorta. *Blood Vessels* 1984; **21:** 180–183.
33. Arrang JM, Devaux B, Chodkiewicz JP, Schwartz JC. $H_3$-receptors control histamine release in human brain. *J Neurochem* 1988; **51:** 105–108.
34. Fredholm BB, Dunwiddie TV. How does adenosine inhibit transmitter release? *Trends Pharmacol Sci* 1988; **9:** 130–134.
35. North RA. Drug receptors and the inhibition of nerve cells. *Br J Pharmacol* 1989; **98:** 13–28.
36. Garbarg M, Trung Tong MD, Gros C, Schwartz JC. Effects of histamine $H_3$-receptor ligands on various biochemical indices of histaminergic neuron activity in rat brain. *Eur J Pharmacol* 1989; **164:** 1–11.
37. Palmer RMJ, Ferridge AG, Moncada S. Nitric oxide release accounts for the biological activity of endothelium-derived relaxing factor. *Nature* 1987; **327:** 524–525.
38. Long CJ, Berkowitz BA. What is the relationship between the endothelium-derived relaxing factor and nitric oxide? *Life Sci* 1989; **45:** 1–4.
39. Rees DD, Palmer RMJ, Schulz R, Hodson HF, Moncada S. Characterization of three inhibitors of endothelial nitric oxide synthase *in vitro* and *in vivo. Br J Pharmacol* 1990; **101:** 746–752.
40. Woolfson RG, Poston L. Effect of $N^G$-L-monomethyl-L-arginine on endothelium-dependent relaxation of human subcutaneous resistance arteries. *Clin Sci* 1990; **79:** 273–278.
41. Bredt DS, Snyder SH. Nitric oxide mediated glutamate-linked enhancement of cGMP levels in the cerebellum. *Proc Natl Acad Sci (USA)* 1989; **86:** 9030–9033.
42. McKinney MC, Bolden CS, Johnson A, Richelson E. Selective blockade of receptor-mediated cyclic GMP formation in N1E-115 neuroblastoma cells by an inhibitor of nitric oxide synthesis. *Eur J Pharmacol* 1990; **178:** 139–143.
43. Mulsch A, Busse R. $N^G$-nitro-L-arginine ($N^5$-(imino-(nitroamino)methyl)-L-ornithine) impairs endothelium-dependent dilations by inhibiting cytosolic nitric oxide synthesis from L-arginine. *Naunyn-Schmiedebergs Arch Pharmacol* 1990; **341:** 143–147.
44. Moore PK, Al-Swayeh OA, Chong NWS, Evans RA, Gibson A. L-$N^G$-nitro-arginine (L-NOARG) a novel L-arginine reversible inhibitor of endothelium-dependent vasodilatation *in vitro. Br J Pharmacol* 1990; **99:** 408–412.
45. Martin W, Villani GM, Jothianandan D, Furchgott RF. Selective blockade of endothelium-dependent and glyceryl trinitrate-induced relaxation by hemoglobin and by methylene blue in the rabbit aorta. *J Pharmacol Exp Ther* 1985; **232:** 708–716.
46. Moritoki H, Tanioka H, Maeshiba Y, Iwamoto T, Ishida Y, Araki H. Age-associated decrease in histamine-induced vasodilatation may be due to reduction of cyclic GMP formation. *Br J Pharmacol* 1988; **95:** 1015–1022.
47. Yan ZQ, Kramer K, Bast A, Timmerman H. The involvement of nitric oxide synthase in the effect of histamine on guinea pig airway smooth muscle *in vitro. Agents Actions* 1994; **41:** C111-112.
48. Carter TD, Pearson JD. Regulation of prostacyclin synthesis in endothelial cells. *News in Physiological Sciences* 1992; **7:** 64–69.
49. Gryglewski RJ, Panczenko B, Korbut R, Grodzinska L, Ocetkiewicz A. Corticosteroids inhibit prostaglandin release from perfused mesenteric blood vessels of rabbit and perfused lungs of sensitized guinea pig. *Prostaglandins* 1975; **10:** 343–345.
50. Vane JR. Inhibition of prostaglandin synthesis as a mechanism of action for aspirin-like drugs. *Nature* 1971; **231:** 232–235.
51. Komori K, Suzuki H. Electrical responses of smooth muscle cells during cholinergic vasodilation in the rabbit saphenous artery. *Circ Res* 1987; **61:** 586–593.
52. Chen G, Suzuki H, Weston AH. Acetylcholine releases endothelium-dependent hyperpolarizing factor and EDRF from rat blood vessels. *Br J Pharmacol* 1988; **95:** 1165–1174.

**Received 1 November 1995; accepted in revised form 12 December 1995**
